# Translational PBPK Modeling of the Protein Therapeutic and CD95L Inhibitor Asunercept to Develop Dose Recommendations for Its First Use in Pediatric Glioblastoma Patients

**DOI:** 10.3390/pharmaceutics11040152

**Published:** 2019-04-01

**Authors:** Nina Hanke, Claudia Kunz, Meinolf Thiemann, Harald Fricke, Thorsten Lehr

**Affiliations:** 1 Clinical Pharmacy, Saarland University, 66123 Saarbrücken, Germany; n.hanke@mx.uni-saarland.de; 2 Apogenix AG, 69120 Heidelberg, Germany; claudiakunz@gmx.de (C.K.); meinolf.thiemann@apogenix.com (M.T.); harald.fricke@me.com (H.F.)

**Keywords:** physiologically-based pharmacokinetic (PBPK) modeling, pediatric PBPK, therapeutic proteins, translational modeling, pediatric drug development, pediatric investigation plan (PIP)

## Abstract

The protein therapeutic and CD95L inhibitor asunercept is currently under clinical investigation for the treatment of glioblastoma and myelodysplastic syndrome. The purpose of this study was to predict the asunercept pharmacokinetics in children and to give dose recommendations for its first use in pediatric glioblastoma patients. A physiologically-based pharmacokinetic (PBPK) model of asunercept in healthy and diseased adults was successfully developed using the available clinical Phase I and Phase II study data. This model was then extrapolated to different pediatric populations, to predict the asunercept exposure in children and to find equivalent starting doses. Simulation of the asunercept serum concentration-time curves in children between 1–18 years of age shows that a dosing regimen based on body weight results in a similar asunercept steady-state exposure in all patients (pediatric or adult) above 12 years of age. For children between 1–12 years, higher doses per kg body weight are recommended, with the highest dose for the very young patients. Translational PBPK modeling is strongly encouraged by regulatory agencies to help with the initial dose selection for pediatric trials. To our knowledge, this is the first report of pediatric PBPK to support the dose selection of a therapeutic protein before its administration to children.

## 1. Introduction

The protein therapeutic asunercept (APG101) analyzed in this study is currently under clinical investigation for the treatment of glioblastoma and myelodysplastic syndrome. Glioblastomas are the most aggressive primary brain tumors [1] and although they are more common in elderly patients, with a median age of 64 years at diagnosis, they also affect children. The incidence of glioblastomas increases with age, from 0.10 per 100,000 in the age group of 0–4 years (average annual incidence rate, diagnosed between 1995 and 2011 in the US, age-adjusted to the 2000 US population), to 0.19 per 100,000 in the age group of 15–19 years, up to 13.09 per 100,000 in the age group of 65–74 years. The 5-year survival rate of the 0–19-year-old patients was 18.2%; for the 65–74-year-old patients the 5-year survival rate was as low as 2.0% [2]. 

In a clinical Phase II study, treatment with asunercept in combination with radiotherapy has shown superiority compared to treatment with radiotherapy alone [3]. The unmet medical need of glioblastoma patients and the benefit of this new treatment option have been recognized by the U.S. Food and Drug Administration (FDA) and the European Medicines Agency (EMA), granting orphan drug designation and EMA PRIME status to asunercept for the treatment of patients with glioblastoma. 

Asunercept is a fully human recombinant glycosylated Fc-fusion protein, consisting of the extracellular domain of the CD95 receptor (CD95/Apo-1/Fas) linked to the Fc part of an IgG antibody. It was designed to interfere with the CD95 ligand (CD95L/FasL) signaling pathway via binding to and neutralization of CD95L. There is mounting evidence that highly invasive malignant glioblastoma express CD95L as an autocrine/paracrine signal that stimulates their invasive growth and migration [4,5]. Asunercept effectively binds to CD95L, blocking the interaction between CD95L and CD95 and thereby exerting an anti-neoplastic effect. Furthermore, asunercept binds to the neonatal Fc receptor (FcRn), conveying protection from lysosomal degradation and increasing the half-life of asunercept. 

The modeling technique used for this analysis, physiologically-based pharmacokinetic (PBPK) modeling, is a valuable tool in state-of-the-art drug development. It is applied to integrate the knowledge gained in the different disciplines of pharmaceutical research, to advance the mechanistic understanding of new drugs and to simulate scenarios that have not yet been tested in the clinics. Due to its modular structure, with separate representations of the properties of the drug on the one hand and the physiology of the modeled individual on the other hand, PBPK modeling is ideally set-up to translate the pharmacokinetics (PK) of a drug observed in an adult population to the situation in children of different age groups. 

The workflow of pediatric PBPK modeling starts with the development and evaluation of a PBPK model for adults, finding the model structure and drug-dependent parameters that accurately describe the behavior of the drug. Then, the physiological parameters of the adult individual are changed to describe the pediatric target population, while the drug-dependent parameters remain unaltered. The PBPK modeling databases used for the generation of individuals provide age-dependent values for anatomical and physiological parameters such as organ volumes, blood flows and tissue compositions, as well as maturation functions for different processes that influence the PK of drugs, allowing for a knowledge-based translation of the anatomy and physiology across the age continuum [6]. 

Pediatric PBPK modeling is strongly recommended by the FDA and EMA to support the initial dose selection for pediatric trials [7,8]. However, while more than 20 pediatric PBPK analyses have been published for small molecules, showing adequate prediction of the pediatric PK in 80% of the studies (neonates ≤ 1 month excluded) [9], to our best knowledge there are no reports of pediatric PBPK modeling to support the dose selection of a therapeutic protein prior to its first administration to children, yet. Considering the vulnerability of the pediatric population, every means available to inform dose finding and selection of blood sampling times should be employed. 

Therefore, the aims of this study were: (i) to develop a whole-body PBPK model of the protein therapeutic asunercept that accurately describes the PK in adult patients; (ii) to translate this model to pediatric populations; (iii) to predict the asunercept PK in pediatric patients; and (iv) to give dose recommendations for children aged 1–18 years that elicit the same exposure of asunercept in children as seen in the adult patient population of the clinical Phase II study.

## 2. Materials and Methods 

### 2.1. Clinical Study Data 

The results of two clinical studies were available for PBPK modeling. Study 1 (APG101_CD_001) was a Phase I first-in-human dose escalation and PK study in 20 healthy volunteers. Asunercept was administered as a single dose (SD) 1-h intravenous infusion, in doses of 0.008, 0.040, 0.200, 1.0, 5.0, 15.0, or 20.0 mg/kg [10]. Study 2 (APG101_CD_002) was a Phase II study in 84 patients with first or second progression of glioblastoma; 58 of these patients received asunercept with concomitant radiotherapy, the remaining patients were treated with radiotherapy only. Asunercept was administered as a weekly (QW) 0.5-h intravenous infusion, in a fixed dose of 400 mg [3]. All subjects gave their informed consent for inclusion before they participated in the studies APG101_CD_001 and APG101_CD_002. Both studies were conducted in accordance with the Declaration of Helsinki. The protocol APG101_CD_001 was approved by the Ethics Committee Berlin (LaGeSo) (ZS EK 13 260/08; EudraCT no. 2008-000130-49); the protocol APG101_CD_002 was approved by the Ethics Committee of the Medical Faculty Heidelberg (AFmu-375/2009; EudraCT no. 2009-013421-42). 

Asunercept serum concentrations below the lower limit of quantification (667 ng/mL for study 1 and 100 ng/mL for study 2) were not considered for this analysis. This included the data of the 2 lowest dosing groups of study 1 (4 subjects). Furthermore, data of study 2 was only used for modeling as long as the patients showed uric acid serum concentrations below 343 µmol/L and were administered with the same batch of asunercept (≥ 3 doses). A preceding nonlinear mixed effects modeling analysis demonstrated that elevated uric acid serum concentrations correlate with a distinct decrease in the clearance of asunercept [11]. As yet, no mechanistic explanation can be offered for this observation. To avoid a bias in the developed PBPK model, 11 patients were excluded due to elevated uric acid serum levels. Furthermore, variability in sialic acid content of the early batches of asunercept was shown to impact the clearance of asunercept, leading to the exclusion of 7 patients due to a change of the asunercept batch after their first or second dose. 5 patients were excluded because they had ≤ 4 asunercept samples taken. No anti-drug antibodies were detected in any of the tested patient sera. 

In total, data of 51 subjects were included for this PBPK analysis, originating from 16 healthy volunteers of study 1 and 35 glioblastoma patients of study 2. The demographics of the different dosing groups are summarized in Table 1. The actual sex, age, weight, height, and ethnicity of every volunteer or patient were used for the individual simulations. Due to the study design and nature of oncological Phase II trials, administration time points and infusion durations of study 2 multiple dose treatment varied from patient to patient. The actual times and durations of drug administration and sampling for each individual were recorded in the case report form and used for the individual simulations. 

### 2.2. Software 

This PBPK analysis was performed using the PK-Sim model environment for mechanistic whole-body PBPK modeling of large molecules (Open Systems Pharmacology Suite 7.2.1, www.open-systems-pharmacology.org). Parameter optimization was accomplished applying the Levenberg–Marquardt algorithm implemented in PK-Sim (optimized parameters are listed in Table 2). Pharmacokinetic parameter analysis and generation of plots were performed with MATLAB R2013b (The MathWorks, Inc., Natick, MA, USA). Sensitivity analysis was carried out in PK-Sim using a relative perturbation of 10% [12] and a sensitivity cutoff of 0.5. A detailed description of sensitivity calculation can be found in the Supplementary Document. 

### 2.3. PBPK Model Building 

Model building was accomplished by implementation and testing of processes known to impact the PK of therapeutic proteins in general and of asunercept in particular. The available clinical data were divided into a training dataset used for model building and parameter optimization, and a test dataset used for model evaluation (see Section 2.4). As training data, all measurements of the 16 healthy individuals of study 1 (0.2, 1.0, 5.0, 15.0, 20.0 mg/kg asunercept, SD), plus data of 19 patients of study 2 (400 mg asunercept, QW) were used. Serum profiles generated with the different batches of asunercept administered in study 2 were evenly distributed to the training and test datasets. Model parameters that could not be informed from literature or in-house experimental research were optimized by simultaneous fitting of all 35 individual simulations of the training dataset to their respective measured serum concentration-time profiles. 

### 2.4. PBPK Model Evaluation 

As test dataset, the asunercept serum concentrations of the remaining 16 patients of study 2 (400 mg asunercept, QW) were used. Model evaluation was accomplished by prediction of the test data and comparison of the predicted to the observed asunercept serum concentration-time profiles. Furthermore, the biological plausibility of the optimized parameter values was investigated and a sensitivity analysis was conducted. To visualize the performance of the final model, all predicted serum concentration values were plotted against their respective observed values in a goodness of fit (GOF) plot. 

For the healthy volunteers of the Phase I trial, the PK blood sampling allows the calculation of pharmacokinetic parameters. Therefore, simulated area under the serum concentration-time curve (AUC_last_), peak serum concentration (C_max_) and serum half-life values of asunercept were calculated for the individuals of study 1 and the resulting values were compared to the clinical observations. As a quantitative measure of the model performance, geometric mean fold errors (GMFEs) were calculated for AUC_last_, C_max,_ and half-life values according to Equation (1):

(1) $$\mathrm{GMFE}\;=\;10^{\;\left(\sum \left|\log_{10}\left(\frac{\mathrm{predicted}\;\mathrm{PK}\;\mathrm{parameter}}{\mathrm{observed}\;\mathrm{PK}\;\mathrm{parameter}}\right)\right|\right)/n}$$

with PK parameter = AUC_last_, C_max_ or half-life value, and *n* = number of individuals. A GMFE value ≤ 2 characterizes an adequate prediction. 

### 2.5. Extrapolation to Children 

To translate the final adult asunercept PBPK model to children, the physiological parameters describing the human body were changed to describe pediatric individuals, while the drug-dependent parameters were kept unaltered. The physiological parameter values for pediatric individuals were taken from the PK-Sim population database. This database contains age-dependent values for organ volumes, blood flow rates, size of the interstitial space, tissue compositions, hematocrit etc., derived from literature such as the report of the International Committee on Radiological Protection (ICRP) [6,13,14,15]. The ontogeny of processes that is not provided in the modeling database has to be implemented based on experimental knowledge, literature information, or assumed to be similar in adults and children (for results on the ontogeny of processes specific to the PK of asunercept please see Section 3.2 and Section 4). 

### 2.6. Prediction of Pediatric Exposure and Development of Dose Recommendations 

To predict the asunercept serum concentration-time profiles in pediatric populations, 18 pediatric subpopulations (50% male), each spanning one year of life from 1 to 19 years of age, were generated using the population database in PK-Sim. These populations were then applied to simulate the asunercept PK of the different age groups. As a reference, an adult population was created according to the full patient demographics of study 2 (67% male, age 20–73 years, weight 50–127 kg, height 151–190 cm, BMI 18-50 kg/m^2^, *n* = 58). 

Since a weekly dose of 400 mg has shown efficacy in adult patients in the clinical Phase II study, the goal of this analysis was to find doses for pediatric patients that generate asunercept serum concentration-time profiles that are as similar as possible to those in study 2. AUC at steady-state (week 15), representing the amount of asunercept in the serum during a dosing interval, was used as the measure of asunercept exposure for dose optimization; however, serum concentration-time profiles and C_max_ values were also compared and assessed. Different dosing approaches were tested, such as body surface area-based dosing and body weight-based dosing. The weekly doses of asunercept for the different age groups were optimized, until the steady-state median AUC of each pediatric population was the same as the steady-state median AUC of the adult reference population of study 2. 

## 3. Results 

### 3.1. Asunercept Adult PBPK Model 

**Figure 1:**
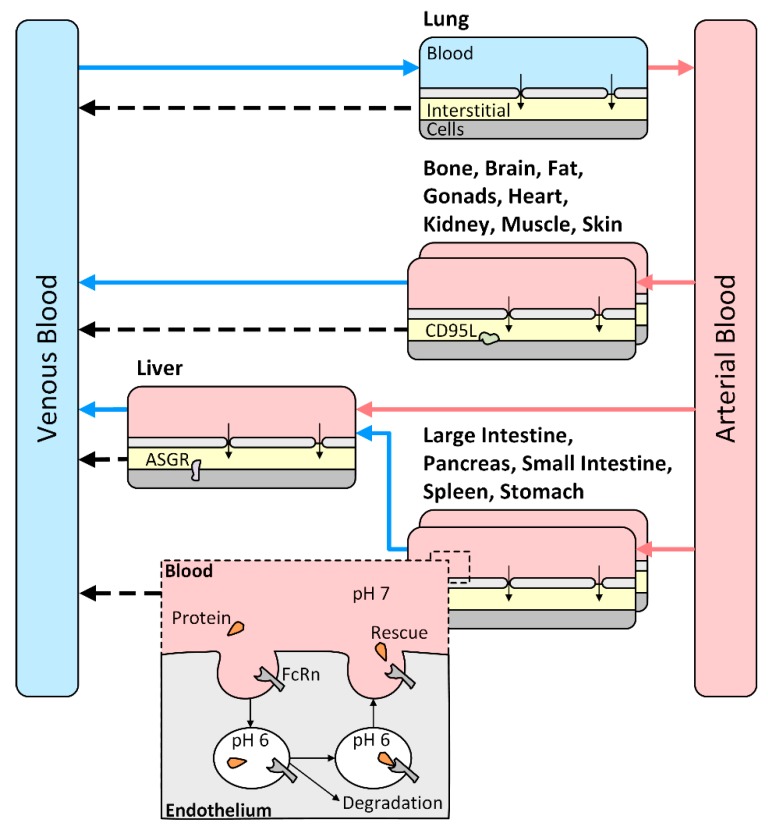
Model structure of the physiologically-based pharmacokinetic model, extended based on [16]. Each of the 15 organ compartments consist of a vascular space (pink or blue), containing blood serum and blood cells, an endothelial barrier (light grey, interrupted by small and large pores), containing lysosomal degradation and rescue thereof by the neonatal Fc-receptor (FcRn), an interstitial space (yellow), connected to the venous blood compartment by organ-specific lymph flows, and a cellular space (dark grey), which is not accessible to large molecules without receptor-mediated uptake. The target of asunercept, CD95L, is mainly expressed on the surface of immune cells, with low levels on neurons, reproductive cells, and other tissues. The asialoglycoprotein receptor (ASGR) is expressed on the surface of hepatocytes and removes glycoproteins with exposed galactose residues from the circulation, which are characterized by the lack of terminal sialic acids.

The general model structure for PBPK modeling of protein therapeutics in PK-Sim is schematically illustrated in Figure 1. It accounts for extravasation of macromolecules from the blood to the interstitial space through small and large pores in the vascular endothelial barrier, lymph flows that return proteins from the interstitial space of the organs to the venous blood pool, as well as the vascular endothelium with its lysosomes as the site of lysosomal breakdown of protein therapeutics and protection from this degradation by the FcRn receptor. Other processes specific to the PK of asunercept were added, namely target binding of asunercept to CD95L as well as an additional clearance pathway via the asialoglycoprotein receptor (ASGR), to obtain the final structural model. 

Asunercept is a glycoprotein carrying 6 N-linked glycans. Mammalian glycans are branched oligosaccharides consisting of N-acetylglucosamine, mannose and galactose components with sialic acid moieties at their outer ends. If the sialic acid is removed or missing to expose the galactose residues beneath, these “asialoglycoproteins” are eliminated from the circulation by ASGR in the liver [17,18]. During the early development of the asunercept manufacturing process, the sialic acid content of the different asunercept batches varied between 0.3 and 0.6 sialic acid residues per glycan. The manufacturing protocol has been improved to consistently produce batches with low variability and a sialic acid content of >0.6 residues per glycan. A nonlinear mixed effects modeling analysis of the combined clinical Phase I and Phase II data found, that batches of asunercept with <0.6 sialic acid residues per glycan show a faster serum clearance of asunercept, probably due to an additional clearance pathway via ASGR [11]. 

The contribution of the asialoglycoprotein receptor to the clearance of asunercept as a function of the sialic acid:glycan ratio of the administered batch was quantified in the nonlinear mixed effects modeling analysis. The result is given in Equation (2):

(2) $${\mathrm{ASGR}\;\mathrm{CL}\;=\;\mathrm{ASGR}\;\mathrm{CL}}_{\mathrm{spec}}\;\times \;(1\;-\;\frac{{\mathrm{sialic}\;\mathrm{acid}:\mathrm{glycan}\;\mathrm{ratio}\;}^{8.03}}{{\mathrm{sialic}\;\mathrm{acid}:\mathrm{glycan}\;\mathrm{ratio}\;}^{8.03}{\;+\;0.378}^{\;8.03}})$$

with ASGR CL = clearance of asunercept by ASGR, ASGR CL_spec_ = maximum ASGR CL for batches with a sialic acid:glycan ratio of zero. 

**Figure 2:**
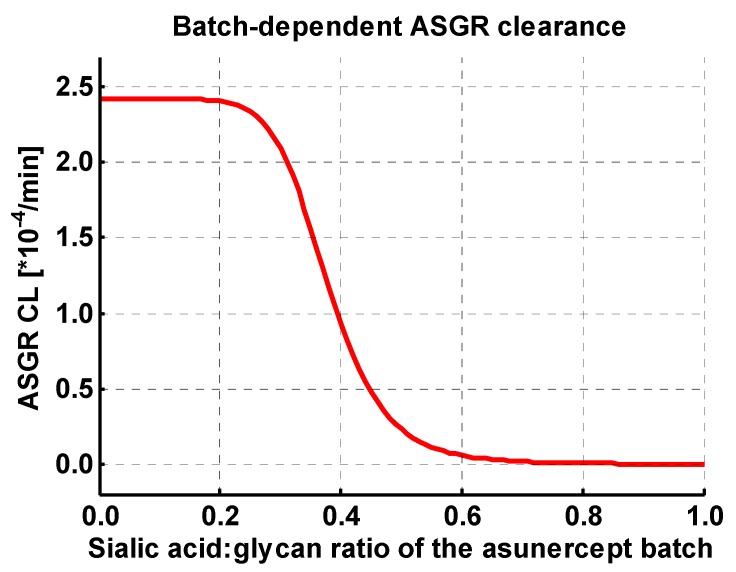
Asialoglycoprotein receptor (ASGR) mediated clearance of asunercept as a function of the sialic acid:glycan ratio of the different asunercept batches. This correlation was identified in a previously conducted nonlinear mixed effects modeling analysis [11] and implemented into the PBPK model. The clearance via ASGR is of no relevance for highly sialylated batches of asunercept with sialic acid:glycan ratios >0.6.

The clearance by ASGR was implemented into the PBPK model according to Equation (2) and one value for ASGR CL_spec_ over all batches was optimized. The resulting ASGR serum clearance as a function of the sialylation of asunercept is illustrated in Figure 2. Although this clearance pathway is negligible for new batches of asunercept, this approach was necessary to correctly describe the data of the two available clinical studies where early batches of asunercept have been administered, and it significantly improved the descriptive performance of the model. 

The final drug-dependent parameters are listed in Table 2. CD95L was implemented into the extracellular membranes of blood, bone, brain, gonads, kidney, and lung cells [5,19,20,21,22,23,24,25], with an assumed relative expression of 1.0 in all tissues and a reference concentration of 1 µmol/L. ASGR was implemented into the extracellular membrane of liver cells with minor expression in other tissues using the PK-Sim gene expression database ArrayExpress profile [26] and a reference concentration of 1 µmol/L. 

**Figure 3:**
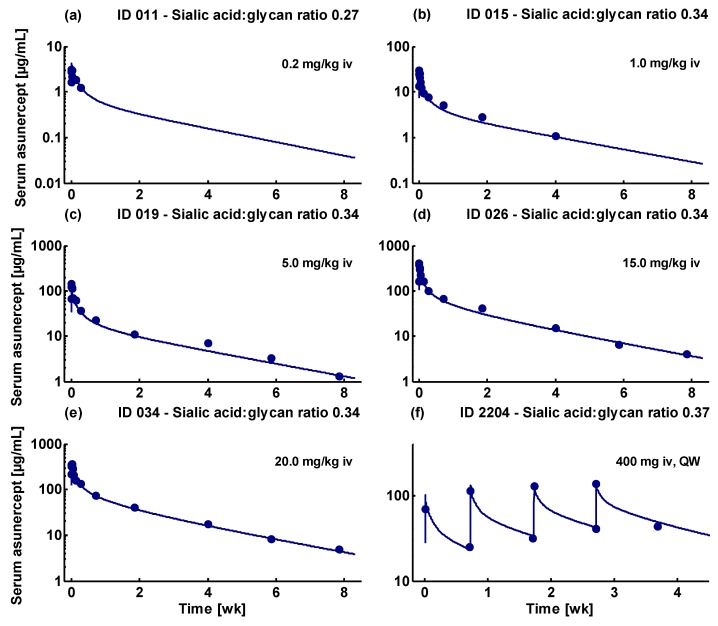
Model performance for intravenous administration of 0.2 mg/kg to 20.0 mg/kg asunercept. Individual simulations are shown as lines, measured asunercept serum concentrations are shown as dots. All presented serum concentration-time profiles are from the training dataset. (**a**–**e**) Asunercept serum concentrations of healthy male volunteers that participated in the Phase I study APG101_CD_001; (**f**) asunercept serum profile of a female glioblastoma patient enrolled in the Phase II study APG101_CD_002. Predicted compared to observed asunercept serum concentration-time profiles of all 51 analyzed individuals are shown in Supplementary Figures S1–S3.

**Figure 4:**
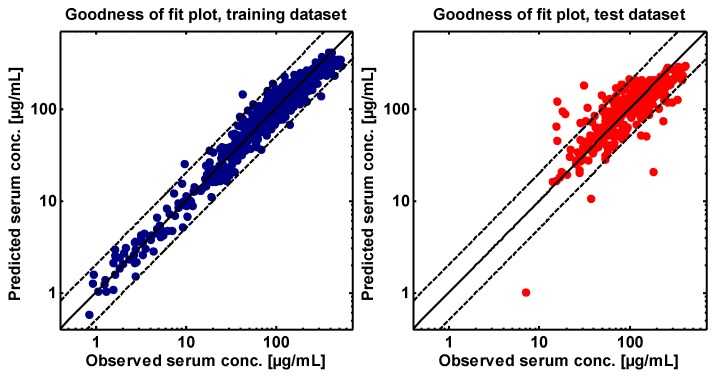
Asunercept goodness of fit plots illustrating the model performance for the training dataset (blue) and the test dataset (red). Shown are predicted compared to observed values of all measured asunercept serum concentrations included in this analysis. The solid lines represent the line of identity; the dashed lines indicate twofold deviation.

The good model performance of the final model is demonstrated by comparison of predicted to observed asunercept serum concentration-time profiles, with examples of every dosing group shown in Figure 3 and simulations of all 51 modeled individuals shown in Supplementary Figures S1–S3. In addition, the goodness of fit plots illustrating the correlation of predicted to observed asunercept serum concentrations, are presented in Figure 4. 

**Figure 5:**
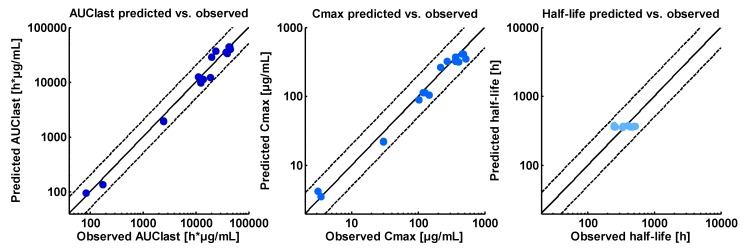
Asunercept predicted versus observed PK parameters illustrating the model performance for the healthy volunteers of study APG101_CD_001 and covering the whole clinically investigated dosing range (0.2, 1.0, 5.0, 15.0, 20.0 mg/kg APG101). **Left**: AUC_last_ values (start of infusion to the last measured concentration, dark blue, *n* = 16), **middle**: C_max_ values (blue, *n* = 16), **right**: half-life values (light blue, *n* = 14). The solid lines represent the line of identity; the dashed lines indicate twofold deviation. The plotted values are listed in Table 3.

The study design of the Phase I study included PK sampling, allowing for the calculation of PK parameters. Plots showing the correlation of predicted compared to observed AUC_last_, C_max_ and half-life values of this clinical trial are presented in Figure 5, covering the whole clinically investigated dosing range. The plotted values are listed in Table 3. 

The sensitivity analysis of the final model was conducted using a sialic acid:glycan ratio of 0.64 for asunercept, representing the batches produced with the new manufacturing method and, therefore, showing no relevant clearance via ASGR. The results are illustrated in Supplementary Figure S5, revealing that for these batches the only parameter the model is sensitive to is the FcRn-asunercept dissociation constant K_d_ in the lysosomal space, which governs the lysosomal degradation of asunercept in the vascular endothelial cells. 

### 3.2. Pediatric Extrapolation 

In addition to the age-dependent adaptation of the anatomical and physiological parameters to describe pediatric populations using the ICRP database, the maturation of the implemented processes that determine the PK of asunercept was investigated. For the expression of CD95L, ASGR, and FcRn, no ontogeny information was given in the PK-Sim database. Therefore, a literature search was conducted but yielded no information on the ontogeny of CD95L. The ASGR1 and ASGR2 proteins are documented to be fully expressed in human fetal liver [27,28]. Furthermore, studies on ASGR in the liver of mice and rats have shown full ASGR binding activity at day 5 after birth for both species [29,30,31]. This corresponds to less than 1 year of age in humans [32,33]. The neonatal Fc receptor protein is also reported to be fully expressed in different fetal human tissues [34]. Therefore, in this analysis CD95L, ASGR and FcRn are assumed to be fully expressed and functional in pediatric patients above 1 year of age. 

### 3.3. Pediatric Dose Recommendations 

**Figure 6:**
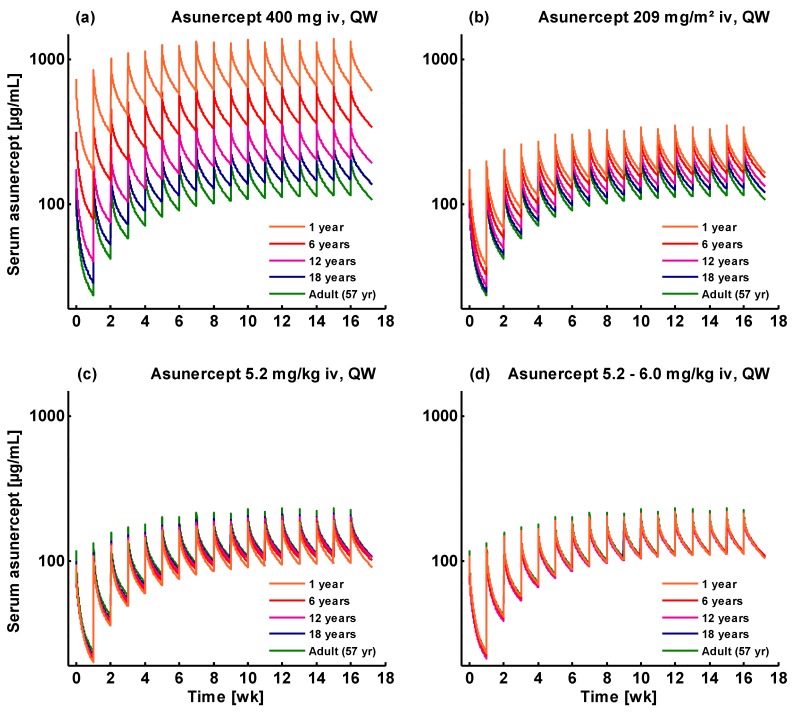
Simulated asunercept serum concentration-time profiles using different dosing approaches in pediatric patients. (**a**) Weekly infusion of a fixed dose of 400 mg; (**b**) weekly infusion of a body surface area normalized dose of 209 mg/m^2^; (**c**) weekly infusion of a body weight normalized dose of 5.2 mg/kg; (**d**) weekly infusion of the recommended doses (see Table 4). Orange: 1-year old patient; red: 6-year old patient; purple: 12-year old patient; blue: 18-year old patient; green: adult Phase II study reference patient. Simulations were conducted using a sialic acid:glycan ratio of 0.64 for asunercept.

To develop dose recommendations for the treatment of pediatric glioblastoma patients in preparation of the pediatric investigation plan, the asunercept serum concentrations in 1- to 18-year old pediatric patients were simulated and compared to those of the adult patients that participated in the Phase II study. Different dosing schemes were tested, showing that dosing based on body weight is the best general approach to achieve a similar exposure in the pediatric populations, as shown in Figure 6. The fixed dose of 400 mg administered in the Phase II study corresponds to 209 mg/m^2^ (mean body surface area of 1.918 m^2^) as well as to 5.2 mg/kg (mean body weight of 77 kg). 

**Figure 7:**
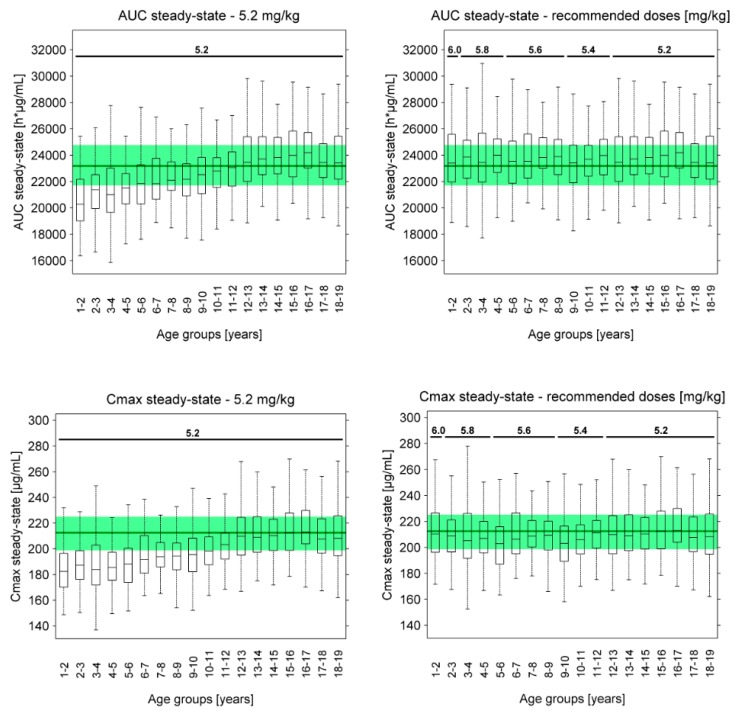
Population predicted asunercept steady-state (week 15) AUC and C_max_ values following administration of 5.2 mg/kg asunercept QW (**left panel**) or of the recommended doses (**right panel**) to children of different age groups. The boxes show population medians, 25th and 75th percentiles, the whiskers represent the most extreme data points not considered outliers. The dark green lines and shaded green areas illustrate the median and 25th to 75th percentile interval of the adult reference population of the Phase II study APG101_CD_002. Simulations were conducted using a sialic acid:glycan ratio of 0.64 for asunercept.

However, using the body weight-based approach, population simulations of 5.2 mg/kg asunercept QW predict lower serum concentrations and lower median AUC and C_max_ values for children <12 years than observed in the adult reference population (see Figure 6c and Figure 7, left panel). As no adverse events were reported in the Phase I dose escalation study, all doses up to 20.0 mg/kg are considered safe and well tolerated in healthy volunteers. Therefore, the recommended doses for patients <12 years were increased until the population medians of the predicted AUCs at steady-state were the same or slightly higher than the AUC of the Phase II adult reference population. Population predicted steady-state AUC and C_max_ values using the recommended doses are presented in Figure 7 (right panel) and the final dose recommendations are listed in Table 4. Population predicted AUC and C_max_ values for the first doses, following administration of 5.2 mg/kg asunercept QW or of the recommended doses, are presented in Supplementary Figure S6. 

## 4. Discussion 

In this study, a whole-body PBPK model of the therapeutic protein asunercept in adult glioblastoma patients was successfully developed, taking into account the lysosomal clearance and rescue thereof by FcRn, target binding to CD95L and an additional clearance via ASGR, which is relevant for several early batches of asunercept. As already observed in a preceding nonlinear mixed effects modeling analysis, the pharmacokinetics of asunercept are dose-linear within the limits of quantification (see Supplementary Figure S4), target mediated clearance did not improve the model performance and was not retained in the final model. This might be due to a very low concentration of CD95L in the body. Binding of asunercept to soluble (cleaved) CD95L as well as to Fc-gamma receptors on the surface of immune cells was assumed to be negligible. The good model performance over the full dosing range and all different batches of asunercept is demonstrated by comparison of predicted to observed serum concentration-time profiles, GOF plots, and, for the Phase I study, by calculation of AUC, C_max_, and half-life values, as well as PK parameter model GMFEs.

For the final model, very few parameters needed to be optimized (CD95L K_d_, CD95L k_off_, ASGR CL_spec_, and the lysosomal FcRn K_d_), with the FcRn-asunercept K_d_ in the lysosomal space being the only fitted parameter the model is sensitive to (for highly sialylated batches of asunercept). This is not surprising, as this value determines the principal clearance pathway of asunercept. In vitro values for the asunercept binding affinities to CD95L and FcRn, determined via surface plasmon resonance, were used during model development. However, optimization of CD95L K_d_, CD95L k_off_ and FcRn K_d_ significantly improved the model performance. The CD95L-asunercept K_d_ value measured in vitro is 7-12 nmol/L, while the optimized value is 1.91 µmol/L with a CD95L reference concentration of 1 µmol/L. A lower K_d_ value for asunercept and a lower expression level of CD95L in the model would most likely better reflect the CD95L in vivo situation, but the higher values significantly improved the model performance. This might indicate a second, less specific and more abundant binding partner of asunercept, that has not yet been identified. The FcRn-asunercept K_d_ value measured in vitro is 12 µmol/L, whereas the optimized value (for binding in lysosomes) is 1.73 µmol/L. This optimized value is closer to the FcRn K_d_ of human IgG in the lysosomal space of 0.63 µmol/L [35] and to other experimental values for Fc-fusion proteins of 2.5–3.6 µmol/L [36]. It results in a predicted asunercept half-life of 15 days (observed half-life 12–15.5 days [10]). Human IgG shows a half-life of 21 days [37], corresponding to its higher FcRn affinity. The fact that different Fc-fusion proteins with identical human Fc-domains show different affinities to FcRn and, therefore, different half-lives (3–23 days [36,38]), as well as the benefit of optimizing the FcRn K_d_ in the lysosomal space have been expertly discussed elsewhere [16]. 

The translation of the model to the pediatric anatomy and physiology was accomplished using age-dependent values for the system parameters of the modeled individuals. The expression and function of CD95L, ASGR, and FcRn were assumed to be fully mature in pediatric patients >1 year of age. In any case, ASGR will not impact the clearance of future asunercept batches produced with the new manufacturing method. Several studies have begun to address the age-dependent factors that influence the pharmacokinetics of protein therapeutics in children [39,40,41,42]. Regarding differences between children >1 year and adults that influence the distribution of protein drugs, a faster extravasation rate was observed in children and has been attributed to proportionally larger organs with sinusoid or fenestrated (leaky) capillaries, such as liver or spleen. Furthermore, a larger extracellular fluid volume of 26% at 1-year of age compared to 18% in adults has been found. Body weight-normalized lymph flow is estimated to be faster in infants than in adults, due to their faster blood flow [41]. The age-dependencies of organ volumes with their respective type of tight or leaky capillaries, the extent of the interstitial space as well as differences in body composition, hematocrit, and blood and lymph flow are taken into account when a pediatric individual is generated using the PK-Sim population database [14,15]. Regarding differences between children >1 year and adults that influence the clearance of protein drugs, a lower concentration of FcRn in the vascular endothelium of children has been proposed as an explanation for the observed faster weight-normalized clearance of many protein drugs in pediatric patients [40,41]. However, although RT-PCR measurements of FcRn expression in rats of different ages have been reported, no ontogeny function could be derived from this data, due to high interindividual and interorgan variability [41,43]. The literature and database search conducted as part of the presented analysis found similar protein levels of FcRn in the few reported human fetal tissues compared to the protein levels in adults [34]. Hardiansyah and Ng recently investigated the expression of FcRn in dependence of age and body weight using a minimal PBPK approach. In agreement with the lower weight-normalized clearance of Bevacizumab and Palivizumab observed in children, they found a negative correlation of FcRn concentration and body weight across different age groups, with an additional decrease of the weight-normalized FcRn concentration with age [42]. Furthermore, in addition to vascular endothelial cells, white blood cells have been described as a major site of FcRn expression and IgG protection [44,45,46,47]. Higher white blood cell counts in children might lead to increased recycling and lower clearance of IgGs. Experimental data on the maturation of FcRn expression and function in human subjects is urgently needed to enable knowledge-based modeling of this important clearance pathway of protein therapeutics. All the above considerations were made concerning children >1 year of age. In younger children, other profound differences have to be taken into account, for example, a much higher capillary surface area, immature capillary endothelium, and high endogenous serum IgG concentrations after birth. Again, these complex developmental changes are not well characterized yet, and further research is required for a better understanding and reliable description. 

The pharmacodynamics (PD) of asunercept have not been considered in this analysis, assuming that the modeled PK directly translates into efficacy. Given that the pharmacologic action of asunercept is mediated via binding to CD95L on the surface of glioblastoma cells, there are two major points to consider: the extravasation of asunercept via the blood-brain barrier (BBB) and the function of CD95L on the tumor cells. The blood-brain barrier is supposed to be impermeable for large molecule therapeutics, but brain tumors, inflammatory processes, and the concurrent radiotherapy during the treatment with asunercept probably facilitate the passage of asunercept across the BBB [48]. Another possibility is the transcytosis of asunercept across the BBB with intermittent binding to FcRn [49]. The pathophysiological conditions relevant for the efficacy of asunercept are assumed to be similar in adult and pediatric glioblastoma patients with regard to the nature of the tumors, the occurrence of inflammatory processes and the concurrent radiotherapy that might temporarily compromise the BBB, enabling the passage of asunercept to the brain. 

Concerning the applied modeling technique, translational PBPK modeling has demonstrated its utility to provide prospective guidance for pediatric starting dose selection of small molecule cancer therapeutics [50]. The FDA reported a total of 12 NDA submissions between 2008 and 2017 applying PBPK modeling for pediatric drug development [51] and the EMA clearly emphasizes the value of PBPK modeling in this field, as well as the need to use all available means to avoid unnecessary trial burden for children [52]. However, the cited reviews also point out the knowledge gaps that need to be addressed in a joint effort, to improve our understanding of pediatric physiology and pharmacokinetics, to share pediatric PK data for model qualification as well as results from pediatric PBPK studies. Although, to the best of our knowledge, this is the first reported prospective pediatric PBPK analysis for a protein therapeutic, many of the pharmacokinetic processes that differ between adult and pediatric populations and that are not well characterized yet, are not relevant for this study. These processes include gastrointestinal changes (intravenous administration), albumin ontogeny (no binding to albumin), changes in hematocrit (no permeation into red blood cells), or changes in glomerular filtration rate (not filtered). Therefore, the maturation and ontogeny of these processes do not impact the presented pediatric model predictions. 

The result of this analysis, showing that body weight-adjusted dosing is the best general approach to achieve equivalent exposure in children and adults, reflects the distribution volume of asunercept, which is restricted to blood serum and interstitial space. The prediction that young children show a lower exposure than adults when administered with the same body weight-based dose of asunercept is also likely accurate, as this phenomenon has been observed in several studies, and was attributed to a faster body weight-normalized clearance of therapeutic proteins in young children and infants (reviewed in [41]). The prediction that a dose increase of 15% per kg body weight (from 5.2 to 6.0 mg/kg) is needed for the 1-year old patients to match the AUC in adults cannot be verified at the moment. Reviewing the dosing regimens of approved protein therapeutics, there is a broad spectrum of age-dependent dosing schemes, ranging from drugs that are given at the same body weight-based dose to all age groups (Daclizumab) up to drugs that are administered at an approximately 100% increased body weight-based dose for the youngest patients (Canakinumab). Tocilizumab is even administered in doses of 12 mg/kg every 2 weeks to patients with systemic juvenile idiopathic arthritis weighing less than 30 kg, compared to 8 mg/kg every 4 weeks to adult patients with rheumatoid arthritis [39]. 

In consideration of the large safety margin of asunercept, the severity of the disease and the dismal prognosis of glioblastoma patients, this translational PBPK analysis suggests higher body weight-based starting doses of asunercept for patients younger than 12 years. The serum concentrations of asunercept in the first pediatric patients need to be closely monitored and will be used to evaluate our model predictions. Above all, the pediatric drug development and clinical research community should try to join forces to speed up the progress of this important and exciting field of research. 

## Figures and Tables

**Table 1:** Dosing groups used for asunercept model development and evaluation.

Dose (mg)	Treatment	*n*	Sex (% male)	Age (years)	Weight (kg)	Height (cm)	SA Ratio	Study Reference
0.2 /kg	iv (1 h), SD	2	100	30–41	66–86	183–187	0.27	APG101_CD_001
1.0 /kg	iv (1 h), SD	2	100	33–44	87–89	178–184	0.34	APG101_CD_001
5.0 /kg	iv (1 h), SD	4	100	28–33	65–94	177–185	0.34	APG101_CD_001
15.0 /kg	iv (1 h), SD	4	100	21–40	64–84	175–183	0.34	APG101_CD_001
20.0 /kg	iv (1 h), SD	4	100	25–44	63–82	178–188	0.34	APG101_CD_001
400	iv (0.5 h), QW	35	62.9	23–73	50–127	151–190	0.30–0.57	APG101_CD_002

iv: intravenous, *n*: number of individuals studied, QW: weekly, SA ratio: sialic acid:glycan ratio of the different asunercept batches administered (for explanation see Section 3.1), SD: single dose. Values given for age, weight, and height are minima and maxima. All study participants were of Caucasian ethnicity.

**Table 2:** Drug-dependent parameters of the final PK-Sim asunercept protein model.

Parameter	Value (95% CI)	Unit	Reference	Description
MW	84082.0	g/mol	APG	Molecular weight
fu	100.0	%	APG	Fraction unbound in serum
Radius (solute)	4.01	nm	calculated	Hydrodynamic radius of the drug
CD95L K_d_	1.91 (±0.28)	µmol/L	optimized	Dissociation constant
CD95L k_off_	9.70 (±3.13)	×10^−5^ /min	optimized	Dissociation rate constant
ASGR CL_spec_	2.43 (±2.98)	×10^−4^ /min	optimized	Normalized first order clearance
FcRn K_d_ (lysosomal)	1.73 (±0.16)	µmol/L	optimized	Dissociation constant
FcRn K_d_ (serum)	999999.0	µmol/L	[16]	Dissociation constant
FcRn k_ass_	0.87	L/(µmol*min)	[16]	Association rate constant
GFR fraction	0.0	-	APG	Fraction of filtered drug reaching the urine

APG: Apogenix in-house result, ASGR CL_spec_: asialoglycoprotein receptor clearance, normalized in the model to batch-specific sialic acid:glycan ratio factors, CI: confidence interval, GFR: glomerular filtration rate. Batch-specific ASGR CL = ASGR CL_spec_ × (1 − (sialic acid:glycan ratio ^8.03^) / (sialic acid:glycan ratio ^8.03^ + 0.378 ^8.03^)).

**Table 3:** Predicted and observed pharmacokinetic parameters of asunercept.

Dose [mg/kg]	ID	AUC_last_ Pred [h*µg/mL]	AUC_last_ Obs [h*µg/mL]	AUC_last_ Pred/obs	C_max_ Pred [µg/mL]	C_max_ Obs [µg/mL]	C_max_ Pred/obs	Half-life Pred [h]	Half-life Obs [h]	Half-life Pred/obs
0.2	010	138	174	0.79	3.5	3.5	1.01	-	-	-
0.2	011	96	84	1.14	4.3	3.1	1.37	-	-	-
1.0	014	1944	2464	0.79	21.9	30.1	0.73	369	340	1.08
1.0	015	1977	2471	0.80	22.3	29.9	0.75	364	258	1.41
5.0	017	12030	18780	0.64	113.4	126.1	0.90	369	513	0.72
5.0	019	11155	13817	0.81	104.2	145.0	0.72	366	248	1.47
5.0	020	9697	12431	0.78	88.8	103.7	0.86	376	249	1.51
5.0	022	12353	11246	1.10	113.5	119.6	0.95	373	419	0.89
15.0	023	34716	37101	0.94	322.6	363.3	0.89	353	332	1.06
15.0	025	37096	24014	1.54	322.3	274.8	1.17	369	420	0.88
15.0	026	33580	39658	0.85	317.9	402.9	0.79	363	472	0.77
15.0	027	28933	20119	1.44	264.7	217.8	1.22	375	401	0.93
20.0	029	40082	45555	0.88	349.0	524.4	0.67	371	496	0.75
20.0	031	43846	42466	1.03	413.1	454.2	0.91	367	424	0.86
20.0	033	43729	44423	0.98	411.9	482.4	0.85	359	437	0.82
20.0	034	40271	44046	0.91	374.1	366.9	1.02	361	437	0.83
GMFE				1.22			1.20			1.24
GMFE range				1.02–1.56			1.01–1.50			1.06–1.51
Pred/obs within 2-fold				16/16			16/16			14/14

AUC_last_: area under the serum concentration-time curve from start of the infusion to the last measured concentration; C_max_: peak serum concentration; GMFE: geometric mean fold error; ID: healthy volunteer identifier; obs: observed; pred: predicted. Individual values are shown.

**Table 4:** Asunercept dose recommendations for pediatric patients.

Pediatric Age Group	Recommended Weekly Dose
1–2 years	6.0 mg/kg
2–5 years	5.8 mg/kg
5–9 years	5.6 mg/kg
9–12 years	5.4 mg/kg
>12 years	5.2 mg/kg

## Supplementary Materials

The following are available online at https://www.mdpi.com/1999-4923/11/4/152/s1, Figure S1: Descriptive model performance for single dose intravenous administration of 0.2 mg/kg to 20.0 mg/kg asunercept to the healthy volunteers that participated in clinical study APG101_CD_001. Individual simulations are shown as lines, measured asunercept serum concentrations are shown as dots. Serum concentration-time profiles are sorted by ascending asunercept dose; Figure S2: Descriptive model performance for multiple dose intravenous administration of 400 mg asunercept QW to the patients in clinical study APG101_CD_002 that were assigned to the training dataset. Individual simulations are shown as lines, measured asunercept serum concentrations are shown as dots. Serum concentration-time profiles are sorted by ascending sialic acid:glycan ratio of the administered asunercept batches; Figure S3: Predictive model performance for multiple dose intravenous administration of 400 mg asunercept QW to the patients in clinical study APG101_CD_002 that were assigned to the test dataset. Individual simulations are shown as lines, measured asunercept serum concentrations are shown as dots. Serum concentration-time profiles are sorted by ascending sialic acid:glycan ratio of the administered asunercept batches; Figure S4: Linear pharmacokinetics of asunercept, observed during the single dose intravenous administration of 0.2 mg/kg to 20.0 mg/kg asunercept to the healthy volunteers that participated in clinical study APG101_CD_001. The dose proportional increase of asunercept serum levels is shown in (**a**) mean asunercept serum concentration-time profiles per dosing group, and (**b**) mean dose-normalized asunercept serum concentration-time profiles per dosing group; Figure S5: Asunercept PBPK model sensitivity analysis. ASGR: asialoglycoprotein receptor, CD95L: CD95 ligand, conc.: concentration, FcRn: neonatal Fc receptor, fu: fraction unbound, kass: association rate constant, Kd: dissociation constant, koff: dissociation rate constant; Figure S6: Population predicted asunercept first dose AUC and C_max_ values following administration of 5.2 mg/kg asunercept (left panel) or of the recommended doses (right panel) to children of different age groups. The boxes show population medians, 25th and 75^th^ percentiles, the whiskers represent the most extreme data points not considered outliers. The dark green lines and shaded green areas illustrate the median and 25th to 75th percentile interval of the adult reference population of the Phase II study APG101_CD_002. Simulations were conducted using a sialic acid:glycan ratio of 0.64 for asunercept.
