# Supplementary material for: Translational PBPK Modeling of the Protein Therapeutic and CD95L Inhibitor Asunercept to Develop Dose Recommendations for Its First Use in Pediatric Glioblastoma Patients
Source: Pharmaceutics. 2019 Apr 1;11(4):152. doi: 10.3390/pharmaceutics11040152 (PMC6523206; doi:10.3390/pharmaceutics11040152)
Supplement: Supplementary file 1 [file pharmaceutics-11-00152-s001.pdf]

pharmaceutics

Supplementary Materials: Translational PBPK Modeling of the Protein Therapeutic and CD95L Inhibitor Asunercept to Develop Dose Recommendations for Its First Use in Pediatric Glioblastoma Patients

Nina Hanke, Claudia Kunz, Meinolf Thiemann, Harald Fricke and Thorsten Lehr *

1. Model Performance: Training Dataset, Phase I Study

Model performance of the final model, demonstrated by comparison of predicted to observed asunercept serum concentration-time profiles of all healthy volunteers that participated in the Phase I study APG101_CD_001 and showed asunercept concentrations above the lower limit of quantification.

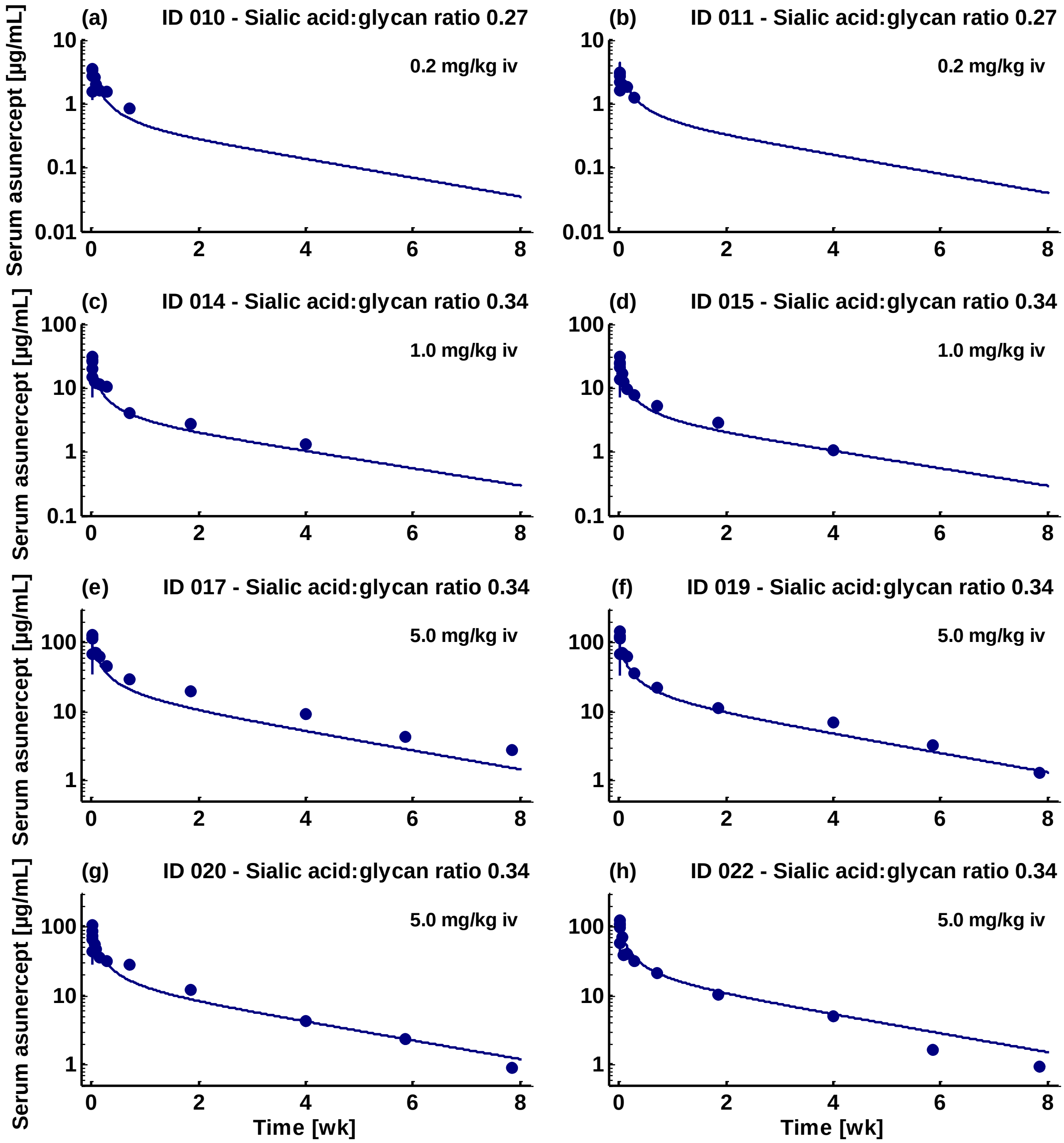

continued...

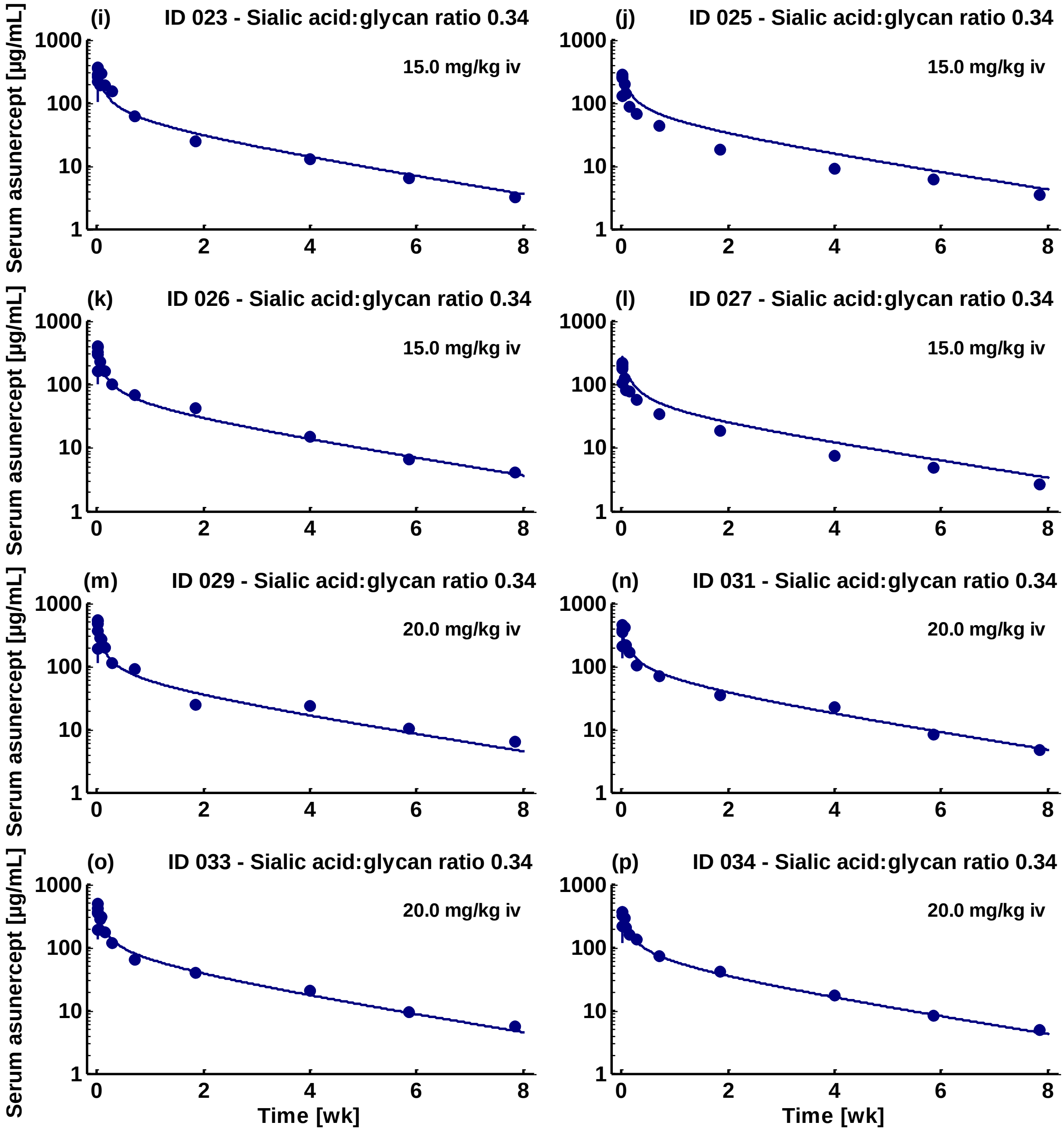

Figure S1. Descriptive model performance for single dose intravenous administration of 0.2 mg/kg to 20.0 mg/kg asunercept to the healthy volunteers that participated in clinical study APG101_CD_001. Individual simulations are shown as lines, measured asunercept serum concentrations are shown as dots. Serum concentration-time profiles are sorted by ascending asunercept dose.

2. Model Performance: Training Dataset, Phase II Study

Model performance of the final model, demonstrated by comparison of predicted to observed asunercept serum concentration-time profiles of the 19 patients that participated in the Phase II study APG101_CD_002 and were assigned to the training dataset.

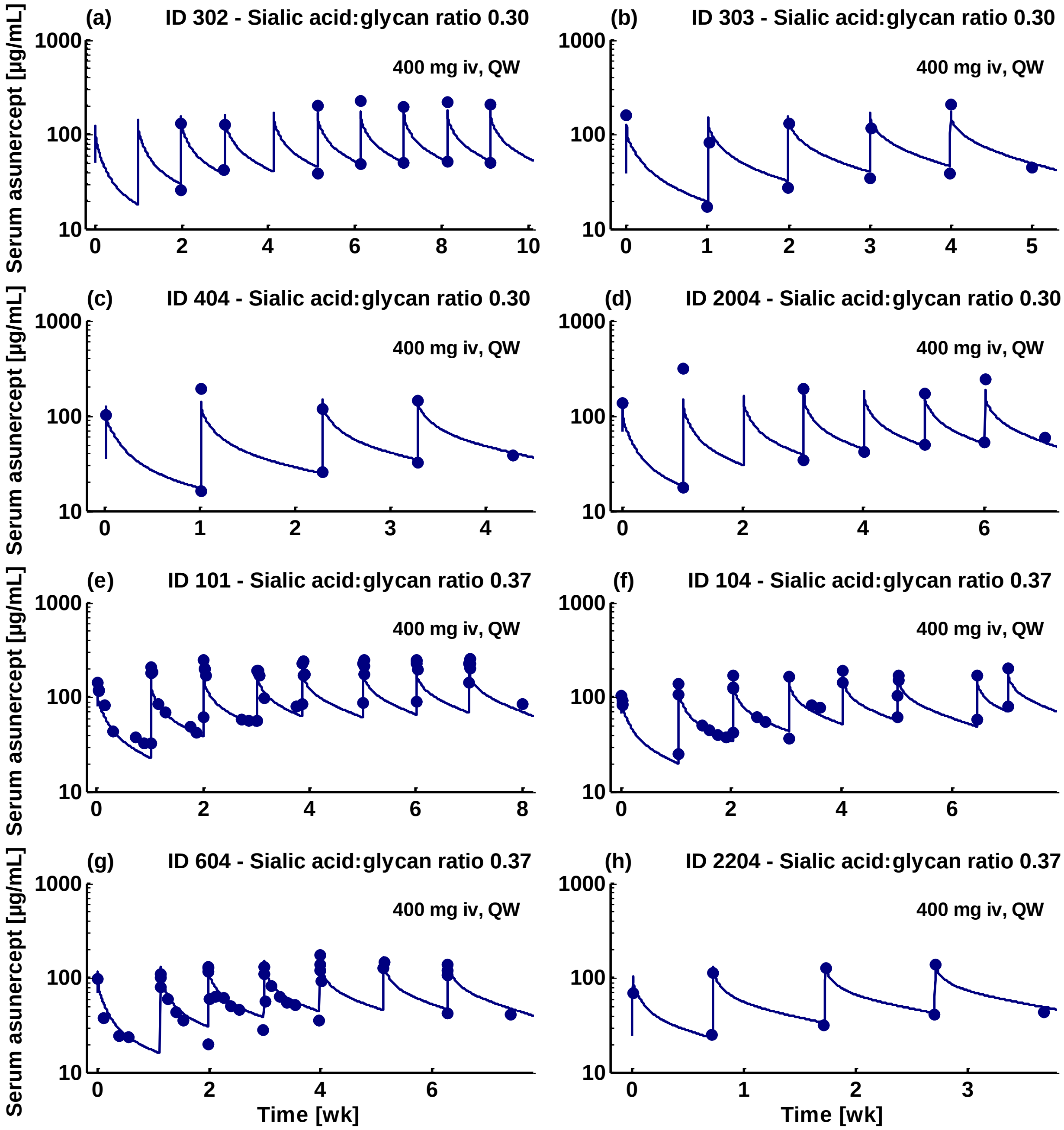

continued...

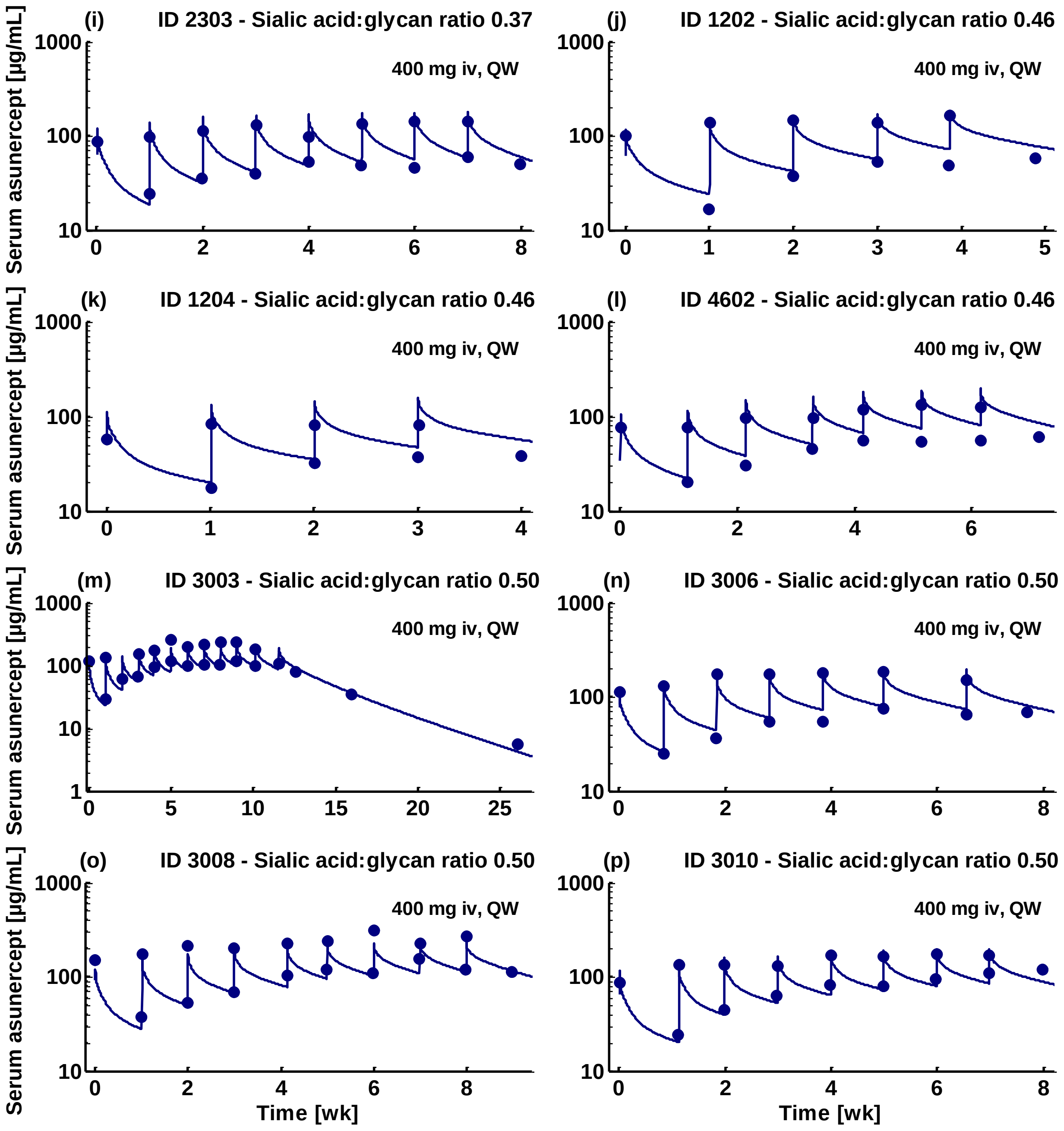

(i) ID 2303 - Sialic acid:glycan ratio 0.37
(j) ID 1202 - Sialic acid:glycan ratio 0.46
(k) ID 1204 - Sialic acid:glycan ratio 0.46
(l) ID 4602 - Sialic acid:glycan ratio 0.46
(m) ID 3003 - Sialic acid:glycan ratio 0.50
(n) ID 3006 - Sialic acid:glycan ratio 0.50
(o) ID 3008 - Sialic acid:glycan ratio 0.50
(p) ID 3010 - Sialic acid:glycan ratio 0.50
400 mg iv, QW
Serum asunercept [µg/mL]
Time [wk]

continued...

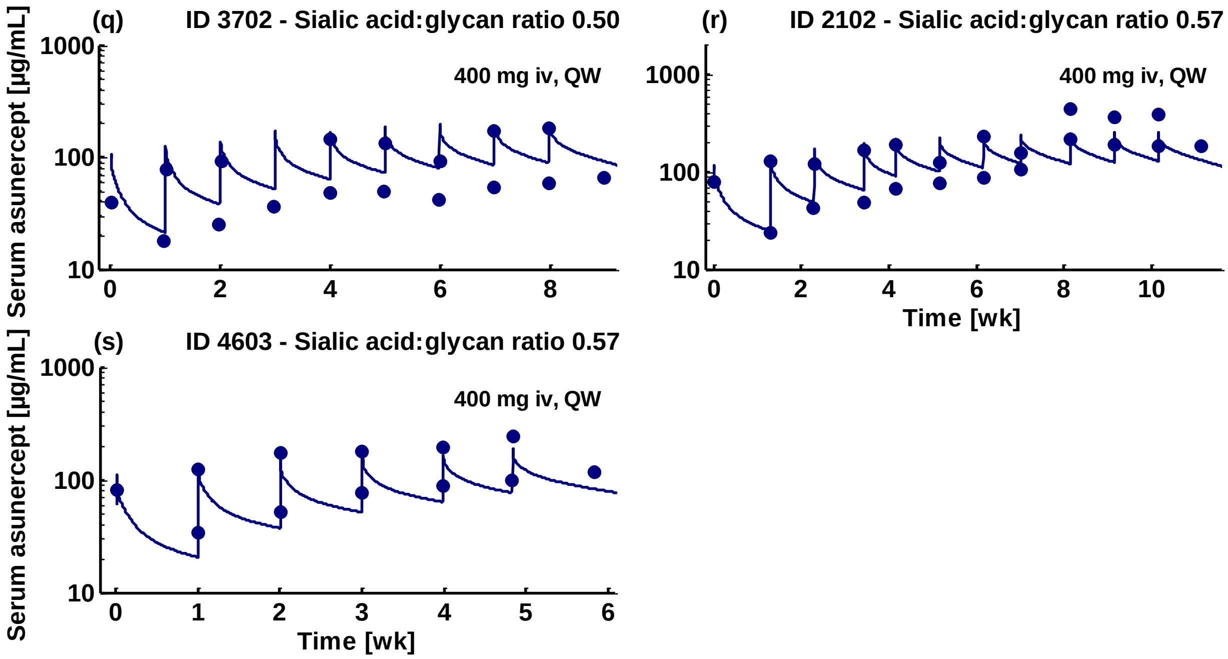

Figure S2. Descriptive model performance for multiple dose intravenous administration of 400 mg asunercept QW to the patients in clinical study APG101_CD_002 that were assigned to the training dataset. Individual simulations are shown as lines, measured asunercept serum concentrations are shown as dots. Serum concentration-time profiles are sorted by ascending sialic acid:glycan ratio of the administered asunercept batches.

3. Model Performance: Test Dataset, Phase II Study

Model performance of the final model, demonstrated by comparison of predicted to observed asunercept serum concentration-time profiles of the 16 patients that participated in the Phase II study APG101_CD_002 and were assigned to the test dataset.

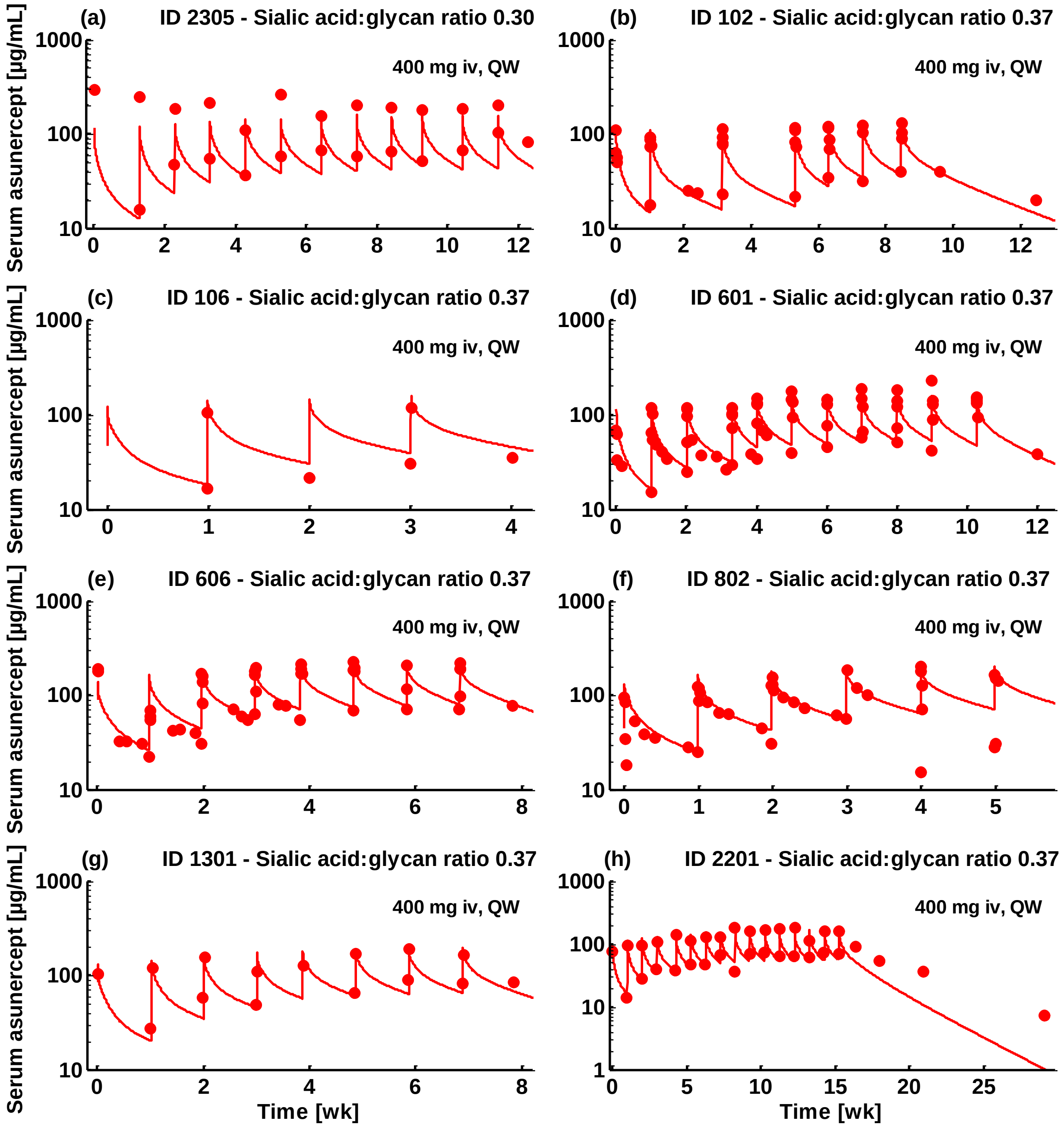

continued...

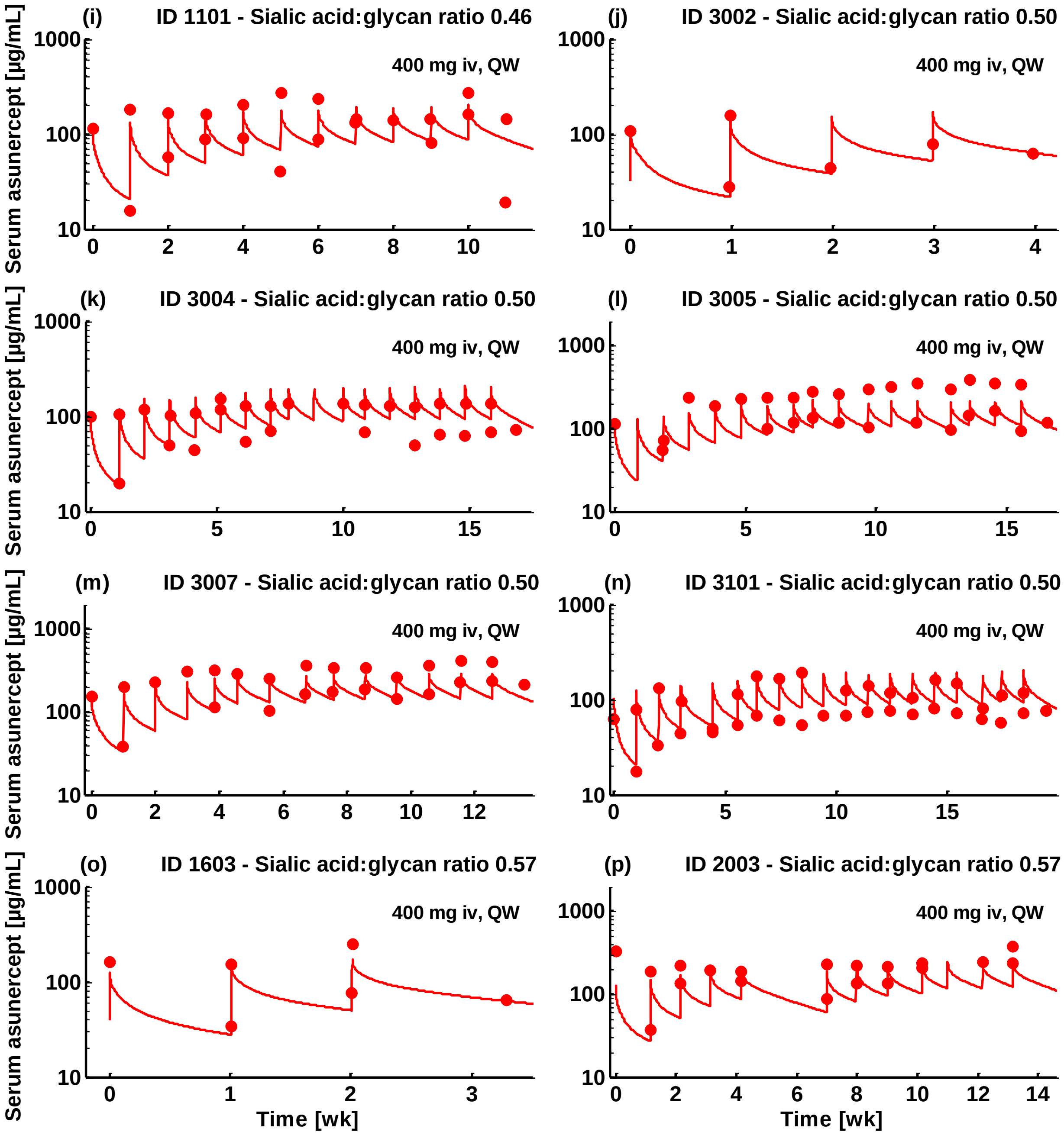

Figure S3. Predictive model performance for multiple dose intravenous administration of 400 mg asunercept QW to the patients in clinical study APG101_CD_002 that were assigned to the test dataset. Individual simulations are shown as lines, measured asunercept serum concentrations are shown as dots. Serum concentration-time profiles are sorted by ascending sialic acid:glycan ratio of the administered asunercept batches.

4. Linear Pharmacokinetics of Asunercept

The mean asunercept serum concentrations per dosing group of the dose escalation Phase I study APG101_CD_001 show linear, dose proportional pharmacokinetics of asunercept for single dose intravenous administration of 0.2 mg/kg to 20.0 mg/kg asunercept to healthy volunteers. The asunercept serum concentrations of the two groups receiving lower doses of 0.008 mg/kg and 0.04 mg/kg asunercept were below the lower limit of quantification.

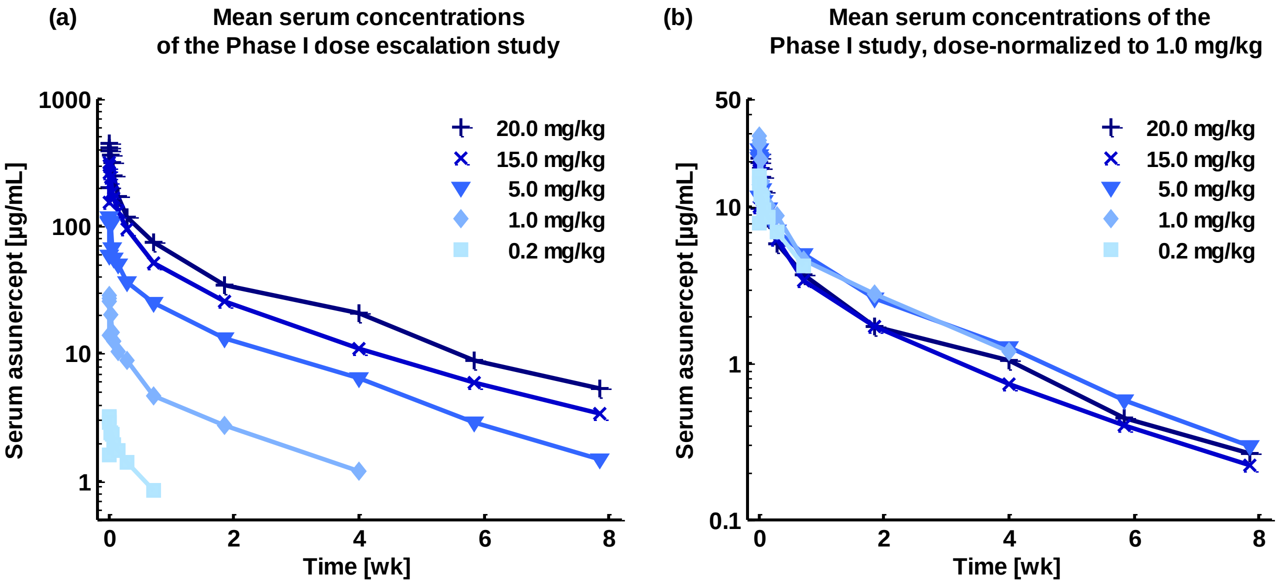

Figure S4. Linear pharmacokinetics of asunercept, observed during the single dose intravenous administration of 0.2 mg/kg to 20.0 mg/kg asunercept to the healthy volunteers that participated in clinical study APG101_CD_001. The dose proportional increase of asunercept serum levels is shown in (**a**) mean asunercept serum concentration-time profiles per dosing group, and (**b**) mean dose-normalized asunercept serum concentration-time profiles per dosing group.

5. Sensitivity Analysis

Sensitivity of the final PBPK model to single parameters (local sensitivity analysis) was calculated, measured as relative change of the AUC at steady-state (week 15) of a 400 mg asunercept QW dosing regimen, using a sialic acid:glycan ratio of 0.64 for asunercept. Sensitivity analysis was carried out with a variation range of 0.1 and number of steps of 2. Using a variation range of 1.0 gave very similar results. Parameters were included into the analysis if they have been optimized (FcRn K_d (lysosomal), CD95L K_d, CD95L k_{off}, ASGR clearance), if they are associated with optimized parameters or if they might have a strong impact due to calculation methods used in the model.

Sensitivity to a parameter was calculated as the ratio of the relative change of the simulated AUC to the relative variation of the parameter around its value used in the final model according to:

$$S = \frac{\Delta AUC}{AUC} * \frac{p}{\Delta p} \quad (1)$$

with S = sensitivity of the AUC to the examined model parameter, ΔAUC = change of the AUC, AUC = simulated AUC with the original parameter value, Δp = change of the examined parameter value, p = original parameter value. A sensitivity of + 1.0 signifies that a 10% increase of the examined parameter value causes a 10% increase of the simulated AUC.

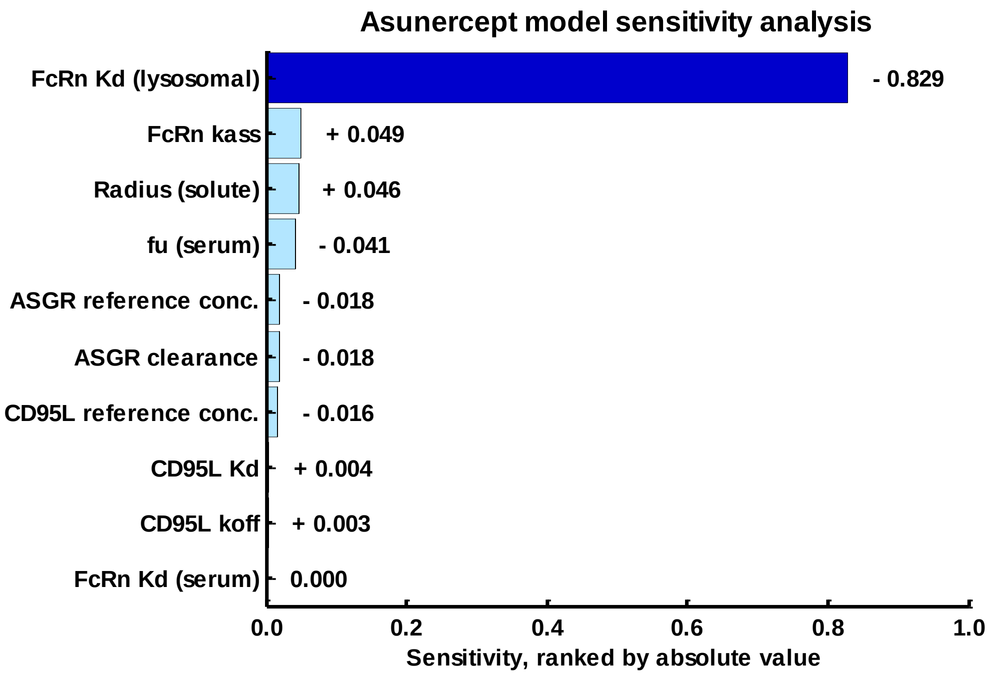

Figure S5. Asunercept PBPK model sensitivity analysis. ASGR: asialoglycoprotein receptor, CD95L: CD95 ligand, conc.: concentration, FcRn: neonatal Fc receptor, fu: fraction unbound, kass: association rate constant, Kd: dissociation constant, koff: dissociation rate constant.

6. Dose Recommendations: Predicted First Dose AUC and C_{max} Values

The asunercept dose recommendations were optimized to generate the same asunercept serum concentrations-time profiles and steady-state AUC values in the pediatric age groups as have been observed in the adult reference population of study APG101_CD_002. The simulated steady-state AUC and C_{max} values using either 5.2 mg/kg asunercept QW for all age groups or the recommended doses are shown in Figure 7 of the main document, the simulated first dose AUC and C_{max} values using these regimens are illustrated below.

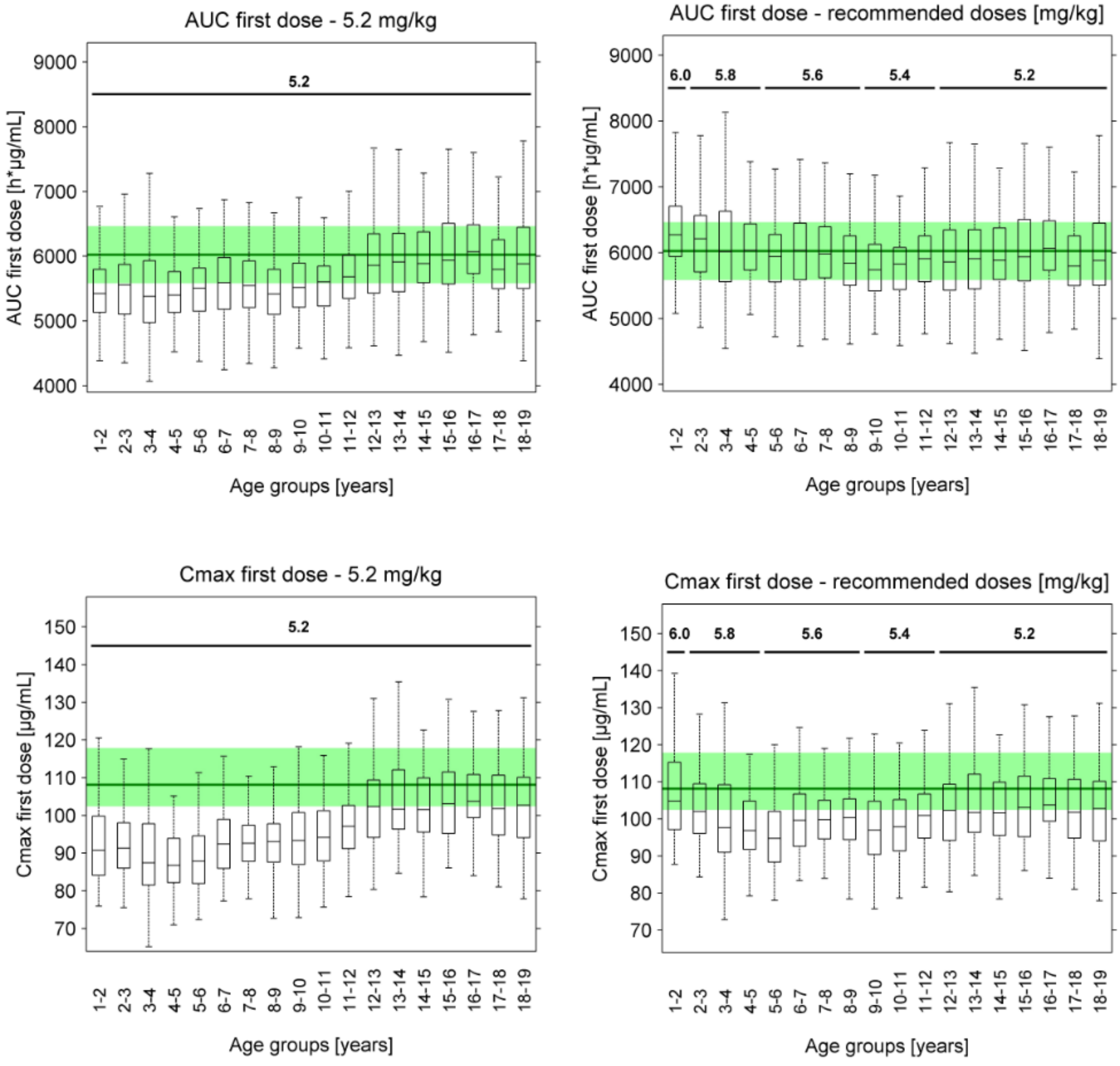

Figure S6. Population predicted asunercept first dose AUC and C_{max} values following administration of 5.2 mg/kg asunercept (left panel) or of the recommended doses (right panel) to children of different age groups. The boxes show population medians, 25th and 75th percentiles, the whiskers represent the most extreme data points not considered outliers. The dark green lines and shaded green areas illustrate the median and 25th to 75th percentile interval of the adult reference population of the Phase II study APG101_CD_002. Simulations were conducted using a sialic acid:glycan ratio of 0.64 for asunercept.

© 2019 by the authors. Submitted for possible open access publication under the terms and conditions of the Creative Commons Attribution (CC BY) license (http://creativecommons.org/licenses/by/4.0/).
